# Enhanced Fringing Field Micro-Moisture Sensor with Elements Optimization

**DOI:** 10.3390/mi17030388

**Published:** 2026-03-23

**Authors:** Xiangrui Meng, Chong Li, Yunlong Lan, Lining Tan, Xiaoxiao Zhang

**Affiliations:** 1 Department of Automation and Measurement, Ocean University of China, Qingdao 266100, China; mxr9697@163.com; 2 School of Automation Science and Electrical Engineering, Beihang University, Beijing 100191, China; dragonlouc@163.com; 3 School of Electronics and Information Engineering, Rocket Force University of Engineering, Xi’an 710025, China; tanlining021@hotmail.com; 4 Institute of Geology and Geophysics, Chinese Academy of Sciences, Beijing 100029, China; zhangxiaoxiao@mail.iggcas.ac.cn

**Keywords:** moisture detection, fringing electric field sensor, sensor optimization

## Abstract

This research demonstrates the principle and optimization methodology to create economic and miniaturized high-resolution micro-moisture sensors. The interdigitated fringe electric field-based moisture measurement principle is firstly investigated to sketch the key parameters of printed circuit board (PCB)-based sensors for further performance optimization. Then, a comprehensive study is conducted to analyze parameter variations with conclusions of suggested design rules to achieve higher measurement sensitivity. Two prototypes are designed and manufactured to validate the proposed theoretical contributions. Water droplets are employed to control the ambient relative humidity, which is adopted as the actual moisture variable in this work. A double-correlated sampling circuit is used for capacitance sensing. Both of them demonstrate a linearity of 1% and sensitivity of 0.1 pF/mg levels, but prototype 2 gains a better batch consistency, which is beneficial for commercialization. Further data analysis suggests that the equivalent input–output sensitivity reaches a level of 1.2403 pF/%RH (relative humidity), which is significantly higher than other types of published interdigitated fringe electric field-type moisture sensors. The optimized prototypes also show advantages of miniaturized size, low cost and high consistency, which can potentially impact the industry applications.

## 1. Introduction

Moisture sensors are widely used in agriculture [1,2], industry [3], medicine [4,5], microelectronics [6], aerospace [7], and other key fields. Based on their sensing mechanisms, these sensors can be classified into resistance type [8], surface acoustic wave type [9], capacitance type [10], impedance type [11], optical type [12], and quartz crystal microbalance type [13], as well as emerging self-powered electrochemical [14] and ion gradient moisture sensors [15]. B. Hosseinzadeh confirmed that the Co-MOF sensing layer integrated with inkjet-printed silver IDEs could significantly enhance the sensitivity and expand the linear detection range of moisture sensors [16]. Jung verified that the graphene oxide layer could greatly improve the humidity sensing performance of multi-frequency surface acoustic wave resonators through comparative experiments [17]. Wang demonstrated that the GOQDs/WS_2_ heterojunction sensing layer markedly enhances the responsivity and shortens the response time of flexible moisture sensors [18]. Kim verified that the carbon black/polyimide composite reduces capacitive moisture sensor hysteresis to 1.80% [19]. Though these types of sensors either show good metrics or scientific innovations, they do not represent a good compromise between performance and manufacturability.

The moisture sensor based on the fringing electric field principle is an important branch due to its high reliability, low cost and faster dynamic responses [20]. For a conventional parallel plate capacitive sensor, it typical constitutes large conductive structure in a horizontal plane. Therefore, the fringe electric field sensor can be applied to the unilateral measurement applications that the parallel plate capacitive-type sensor is difficult to handle [21]. A.V. Mamishev is considered to be the first scholar to investigate the fringe electric field sensor [22]. He explained that the interdigitated fringe electric field sensor has stronger measurement sensitivity than the conventional capacitive structure of the sensors [23]. Li proposed that optimizing the interdigital electrode structure and designing a novel heating layer balances the sensitivity and dynamic response of capacitive moisture sensors, improving heat transfer uniformity [24]. While the performance of this electric field principle is improved, how to manufacture and produce this kind of sensor is another difficult problem in the research of moisture sensors based on the fringe electric field principle.

In order to develop a high-performance and low-cost interdigitated fringe electric field moisture sensor, printed circuit board (PCB) technology has played an important role. Compared with MEMS processes, PCB-based manufacturing has the advantages of much higher yield rate, shorter lead time, lower cost and being easier for assembly [25]. Jiao proved that the interdigitated fringe electric field sensor can effectively improve its performance by optimizing key parameters and improve its stability to ambient moisture, but there is no superior detection circuit designed to read the signal [26]. Based on printed circuit board technology, Utpal Sarma designed three fringing field capacitive soil moisture sensors, but the selection of capacitive parameters can be further improved [27]. Robert N. Dean developed the fringe electric field sensor for moisture sensing by using PCB processing technology, and demonstrated the promising potential of this type of sensor for its good linearity and sensitivity [28]. Petre et al. verified the feasibility of low-cost, high-yield fabrication of fringing field sensors via standard PCB processes [29]. In some results, the correlation coefficient R between the equivalent sensitive capacitance and the moisture content of corn kernels is 0.872, and the sensor has high sensitivity [30]. Tarikul Islam applied the fringe electric field sensor to the liquid level measurement, and deeply analyzed the influence of electrode ratio on the performance of the sensor [31]. Recent research has advanced PCB-fabricated interdigitated capacitive sensors, with improved detection precision and linear response, further validating the low-cost and high-performance advantages of PCB-based interdigitated fringe electric field moisture sensors [32,33].

Though the performance of the fringe field moisture sensor is promising, it lacks a comprehensive study to give guidelines for sensor optimization. In this paper, the principle of sensing based on fringe field is introduced and its main components are disassembled and analyzed. Then, a finite elements analysis-aided optimization is performed to maximize the performance. The remainder of the paper gives the details.

## 2. Fringing Electric Field Effect and Interdigitated Electrode Structure

### 2.1. Capacitance and Its Fringing Electric Field Effect

The essence of moisture detection is that it changes in ambient moisture, leading to changes in the corresponding characteristics of the moisture sensor. Capacitance is a measure of the ability of two conductors to store electric charge, and its characteristics vary with ambient moisture levels. When there is a certain potential V difference between two isolated conductors in space, free charge accumulates on the conductor, and the ratio of the accumulated charge Q to the potential difference V is the capacitance C:

(1) $$C=\frac{Q}{V}$$

The parallel plate capacitor consists of two parallel conductive electrode plates. The opposite electrode plate has a length of $l_{1}$, a width of $l_{2}$, and an area of S. The distance between the plates is d, and the dielectric constant between the plates is ε. According to Maxwell’s equations in electromagnetic theory, the calculation formula of parallel plate capacitance can be obtained. Regardless of the edge effect, the capacitance of parallel plate capacitors is as follows:

(2) $$C=\frac{\varepsilon S}{d}$$

However, the actual situation is that the plate area of the capacitor is limited, and charge carriers cannot extend to an infinite plane and instead accumulate at the electrode edges. At this time, the electric field lines on the edge of the plate are radiated to the outside of the plate in an arc shape. This is the fringing electric field effect of the capacitor which causes the surface charge of the plate to be no longer completely uniform, so that Equation (2) is not completely true. Therefore, its form should be rephrased as follows:

(3) $$C^{\prime }=\frac{2\varepsilon l_{1}}{d\pi }[ln(e^{-\frac{\pi l_{2}}{2d}}+e^{\frac{\pi l_{2}}{2d}})+ln2]$$

This has a negative impact on sensing measurements based on the principle of parallel plate capacitance, such as degraded linearity. When the ratio of the plate area to the substrate thickness of the parallel plate capacitor continues to decrease, the fringing electric field effect of the capacitor becomes increasingly pronounced. These changes cause the parallel plate capacitor to evolve into a capacitor that operates based on the fringing electric field effect.

### 2.2. Interdigitated Electrode Structure and Moisture Detection

**Figure 1:**
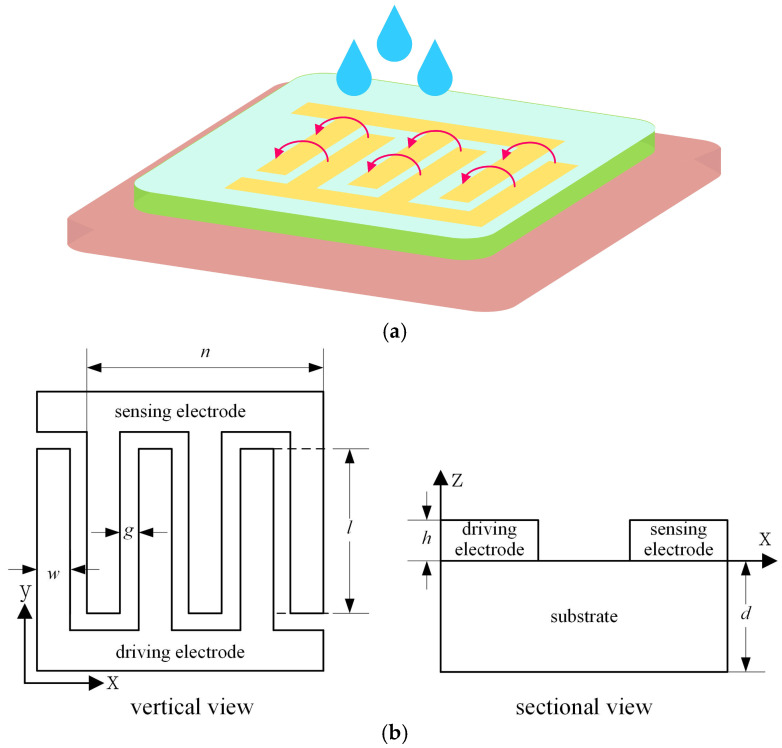
Interdigitated electrode-based moisture sensing principle and structural schematic. (**a**) Moisture detection based on interdigitated electrodes. (**b**) Structural parameters of interdigitated electrodes.

Due to the high signal strength and sensitivity, we choose the interdigitated structure to design the capacitance sensor, and realize the moisture detection based on the fringing electric field effect, with the principle of moisture detection illustrated in Figure 1a.

The interdigitated electrode structure is shown in Figure 1b; it consists of three parts. They are the driving electrode, the sensing electrode and the substrate, respectively. In Figure 1b, w is the electrode width, g is the distance between the driving electrode and the sensing electrode, n is the number of electrode pairs, h is the electrode thickness, d is the substrate thickness, l is the length of the overlapping area of the adjacent electrodes, and ε is the substrate material dielectric constant. The relationship among the electrode structural parameters l, h, and g is given by the following:

(4) $$lh>>g^{2}$$

Therefore, the metal coverage of the sensor α can be characterized by the electrode width w and the electrode spacing g:

(5) $$\alpha =\frac{w}{w+g}$$

According to reference [30], the equivalent capacitance $C_{S}$ of the interdigitated electrode structure can be approximated as follows:

(6) $$C_{S}=\frac{n\varepsilon_{S}lh\gamma }{g}$$

where $\varepsilon_{S}$ is the dielectric constant of the medium between electrodes, and γ is the fringe field effect factor.

It can be known from Equation (6) that, when the ambient moisture changes, the dielectric constant of the medium between electrodes $\varepsilon_{S}$ will change accordingly. This eventually leads to the change in the equivalent capacitance of the interdigitated electrode structure. This is the principle of the interdigitated fringe electric field sensor for moisture detection.

### 2.3. PCB Structure for Interdigitated Electrode

The PCB processing technology of the interdigitated structure design is introduced.

PCB processing technology has the characteristics of short production cycle, low production cost and stable quality. It has the processing capability to realize the interdigitated electrode structure. The PCB structure of the two-layer board consists of two solder mask layers, two copper layers, and one substrate layer, where the solder mask is a polymeric coating used to limit solder flow during the reflow process. The substrate layer represents the shape outline of the entire PCB, and it can isolate the electrical connection between the upper copper layer and the lower copper layer. There are two types of substrate layers: rigid substrates and flexible substrates. Our design uses a rigid substrate with a typical thickness of about 1.5 mm. The copper layer is used to design the copper traces or lay the copper sheet. We design copper wires in the copper layer to realize the interdigitated capacitor structure. The thickness of the copper wire ranges from 0.035 mm to 0.07 mm, and the minimum width that can be processed is 0.153 mm. This layer prevents solder from adhering to non-soldering areas of the PCB. This facilitates the realization of reflow soldering process technology, which can improve soldering efficiency. This layer of solder mask green oil can also provide insulation and anti-oxidation. The minimum plated vias have an inner diameter of 0.2 mm and an outer diameter of 0.3 mm.

## 3. Key Performance Indicators of Sensors

The performance indicators of the sensor concerned in this manuscript are: the signal strength of the sensor, the capacitive response sensitivity and the relative capacitive response sensitivity. They are defined as follows:

### 3.1. The Signal Strength of the Sensor

In this paper, the initial capacitance of the fringing electric field sensor is used to characterize its signal strength. The initial capacitance of the sensor is the equivalent capacitance value $C_{air}$ of the sensor in the air medium.

### 3.2. The Capacitive Response Sensitivity

Capacitive response sensitivity is defined as the ratio of the change in the equivalent capacitance value of the sensor to the change in the dielectric constant of the detection area. Its form is as follows:

(7) $$\delta C=\frac{\Delta C_{S}}{{\Delta \varepsilon }_{S}}$$

where $\Delta C_{S}$ is the equivalent capacitance change in sensor output (pF), and ${\Delta \varepsilon }_{S}$ is the change in the relative dielectric constant of the medium in the sensor sensitive region (dimensionless).

### 3.3. The Relative Capacitive Response Sensitivity

The relative capacitive response sensitivity is defined as the ratio of the capacitive response sensitivity δC of the sensor to the initial capacitance $C_{air}$ of the sensor. Its form is as follows:

(8) $$\delta =\frac{\delta C}{C_{air}}$$

The initial capacitance value of the sensor determines whether the sensor is feasible. In order to complete the moisture detection, the initial capacitance of the sensor needs to be increased as much as possible. The capacitive response sensitivity represents the sensitivity of the sensor to changes in external media. Under the same external medium change, the sensor with high sensitivity has a larger output, and it has a better signal-to-noise ratio. High-sensitivity sensors facilitate signal processing. The relative capacitance response sensitivity is normalized on the basis of the capacitance response sensitivity and it can better reflect the effectiveness of the sensor design. All three metrics are positive performance indicators, where higher values correspond to better sensing performance. The work of this paper is guided by them to optimize the design of sensor structure parameters.

## 4. Structural Parameter Optimization for Design of Sensor

The fringe electric field sensor has problems, such as the fact it has difficulty in calculate accurately and its inherent nonlinear characteristics. These problems limit the application of such sensors. Finite element analysis methods can decompose the solution domain of a complex problem into multiple discrete interconnected sub-domains called finite elements. The approximate solution is obtained in each finite element, and finally, the solution of the solution domain is deduced based on specific conditional criteria. This section describes the principle and workflow of finite element analysis in COMSOL Multiphysics 5.6. Then, it explores the influence of the main structural parameters of the interdigitated fringe electric field sensor on its key performance indicators. The work in this section provides optimization directions for sensor prototype designs.

### 4.1. Electric Field Construction of Fringe Electric Field Sensor in COMSOL Multiphysics

COMSOL Multiphysics completes the analysis and calculation of the electrostatic module based on Maxwell’s equations and constitutive relations. Maxwell’s equations and constitutive relations are as follows:

(9) ∇×E=0

(10) ∇×D=ρ

(11) D=εE

where E is the electric field strength, D is the electric displacement vector, ε is the dielectric constant of the medium, and ρ is the charge density.

According to Equation (9), the electrostatic field is an irrotational vector field. According to the vector identity, the irrotational vector field can be expressed as the gradient of the scalar field. This scalar field is the electric potential φ; its form is as follows:

(12) E=−∇φ

Based on the above equations, the electric potential, electric field, distribution of electric charge, and electrostatic energy storage can be calculated in the model. From these data, the equivalent capacitance of the sensor can be calculated, and the calculation form is as follows:

(13) $$C=\frac{2E_{\varepsilon }}{V^{2}}$$

where $E_{\varepsilon }$ is the electrostatic energy stored in the sensor (J), and V is the applied electric potential across the sensor electrodes (V).

### 4.2. Optimization of Structural Parameters Based on Simplified Sensor Model

The work of this manuscript is based on the simplified model of the interdigital fringe electric field sensor, and then the simulation analysis is carried out. This simplified model only contains a single pair of electrodes, including the driving electrode, sensing electrode, substrate and the dielectric domain surrounding the sensor. For mesh division in the simulation, an adaptive strategy is adopted, which determines the fineness of mesh division according to the scale of different regions: finer meshing is applied at geometry boundaries, smaller volumes and narrower faces, while relatively sparse meshing is used in other regions. This strategy can well balance the calculation accuracy and simulation speed of the model.

The parametric sweep function can be provided by COMSOL Multiphysics software. This function can analyze the influence of the main structural parameters on key performance indicators. The parametric sweep function can set a structure parameter to change with a certain step size within a specified range. It automatically calculates simulation results, resulting in a data set of key performance indicators that vary with structural parameters. The optimization direction of each parameter can be obtained by analyzing the data set.

In order to further improve the calculation accuracy of the sensor’s equivalent capacitance, capacitance calculations are performed under both external boundary insulation and conduction conditions. For the insulation condition, the electric field lines are distributed parallel to the boundary; for the conduction condition, the electric field lines are distributed perpendicular to the boundary. The equivalent capacitances of the sensor under these two boundary conditions are calculated separately and then averaged, and the average value is used as the final calculation result of the sensor’s equivalent capacitance. All subsequent capacitance calculations of the sensor in this manuscript adopt this method.

#### 4.2.1. Influence of Metal Coverage on Key Performance

**Figure 2:**
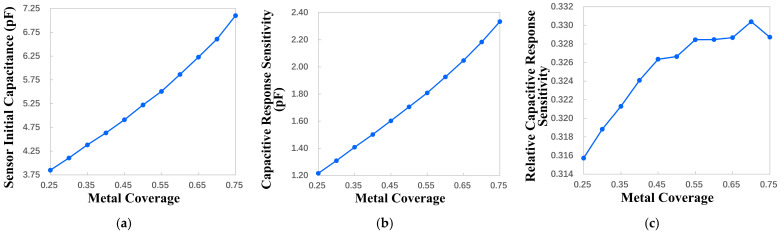
The influence of metal coverage variation on key performance indicators. (**a**) Sensor initial capacitance change. (**b**) Capacitive response sensitivity change. (**c**) Relative capacitive response sensitivity change.

The metal coverage is defined as shown in Equation (5). The increase in the metal coverage leads to an increase in the electrode volume and a decrease in the electrode spacing. This enhances the electrode’s ability to store charge, which also increases the sensor’s equivalent capacitance. The variation range of the metal coverage is set to be 0.25 to 0.75 with a step size of 0.05. Through the parametric sweep, the relationship between the key performance indicators and the metal coverage is obtained, as shown in Figure 2. According to Figure 2, with the increase in the metal coverage, the initial capacitance and capacitance response sensitivity of the sensor are improved. This is consistent with the theoretical analysis results. The relative capacitive response sensitivity increases slightly at a metal coverage of 0.25 to 0.45, and then remains essentially constant at 0.45 to 0.70, but decreases in the range of metal coverage from 0.70 to 0.75. It can be seen that the improvement of the metal coverage brings a limited improvement in the overall performance of the sensor. However, the increase in the metal coverage leads to the use of more metal materials in production and processing, and the production cost will increase.

For the above reason, the sensitivity, signal strength and cost are considered comprehensively, and the metal coverage is chosen to be 50%.

#### 4.2.2. Influence of Substrate Thickness on Key Performance

**Figure 3:**
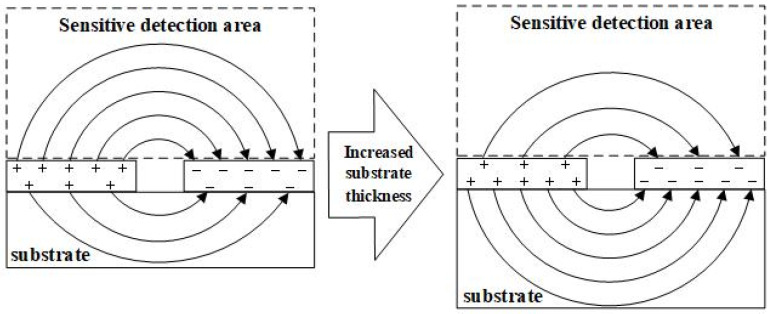
Schematic diagram of the change in electric field line distribution with the increase in substrate thickness.

The electric field lines generated by the interdigitated electrode structure are distributed in arcs above and below the electrodes, as shown in Figure 3. The electric field lines above the electrodes can sense the physical properties of the measured object and thus change, and this area is the sensitive detection area of the sensor. The electric field lines under the electrodes are distributed in the substrate of the sensor, and this area is the non-sensitive detection area of the sensor. Theoretical analysis shows that, as the basic thickness increases, more electric field lines pass through the substrate. The dielectric constant of the substrate is usually higher than that of air, which ultimately results in an increase in the initial capacitance of the sensor. The substrate area is the non-sensitive detection area of the sensor, and the increase in the thickness of the substrate makes more electric field distributed in the non-sensitive detection area. The increase in substrate thickness results in a decrease in the capacitive response sensitivity of the sensor.

**Figure 4:**
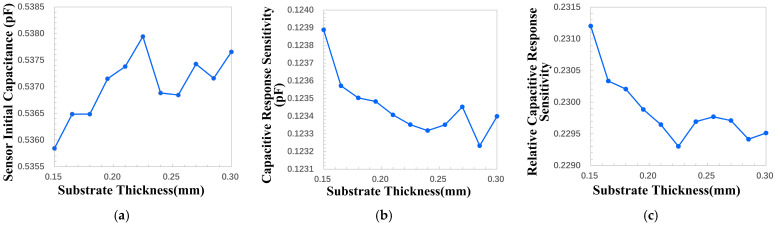
The influence of substrate thickness variation on key performance indicators. (**a**) Sensor initial capacitance change. (**b**) Capacitive response sensitivity change. (**c**) Relative capacitive response sensitivity change.

The minimum value of substrate thickness that can be processed in this paper is 1.5 mm, and the variation range of the substrate thickness is set to be 1.5 mm to 3 mm with a step size of 0.01 mm. Through the parametric sweep, the relationship between the key performance indicators and the thickness of the substrate is obtained, as shown in Figure 4. It can be seen that, with the increase in the thickness of the substrate, the initial capacitance of the sensor increases, and the capacity for increase gradually decreases. Meanwhile, the capacitive response sensitivity decreases with the increase in substrate thickness. As a result, when the substrate thickness is 1.5 mm (the minimum processable thickness via standard PCB manufacturing), the relative capacitive response sensitivity reaches its maximum value. These results are consistent with the theoretical analysis results. The increase in substrate thickness consumes more substrate material and results in a larger sensor volume. It has adverse effects in terms of economic efficiency and portability.

For the above reason, the thickness of the substrate should be minimized, and the minimum thickness of the substrate that can be processed in this paper is 1.5 mm.

#### 4.2.3. Influence of Relative Dielectric Constant of Substrate on Key Performance

**Figure 5:**
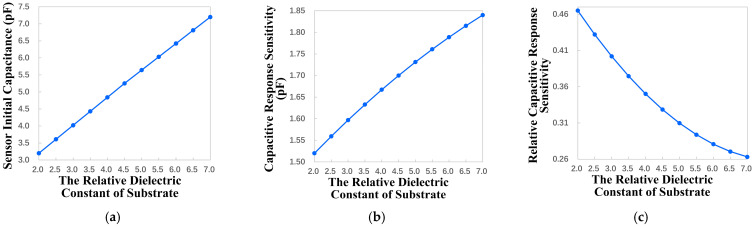
The influence of the relative dielectric constant of the substrate variation on key performance indicators. (**a**) Sensor initial capacitance change. (**b**) Capacitive response sensitivity change. (**c**) Relative capacitive response sensitivity change.

The relative dielectric constant of the substrate also affects the key performance indicators of the sensor by affecting the electric field strength in the effective sensitive detection area of the sensor; it is similar to the effect of the thickness of the substrate. The variation range of the relative dielectric constant of the substrate is set to be 2 to 7 with a step size of 0.5. Through the parametric sweep, the relationship between the key performance indicators and the relative dielectric constant of the substrate is obtained, as shown in Figure 5.

According to the results, as the relative dielectric constant of the substrate increases, the initial capacitance and capacitance response sensitivity of the sensor both increase, but the relative capacitance response sensitivity decreases. As the relative dielectric constant of the substrate increases, there are both positive and negative effects on sensor performance.

For the above reason, the design of this sensor can use normal substrate material, such as FR-4 glass fiber board, which has good economic benefits.

#### 4.2.4. Influence of the Number of Electrode Pairs on Key Performance

In the case of a fixed sensor detection area and metal coverage, this manuscript explores the effect of the number of electrode pairs of electrodes on key performance, and its essence is to explore the effect of the uniformity of the sensor’s interdigitated electrodes on key performance. Under the condition of fixed detection area and metal coverage, as the number of electrode pairs of the sensor increases, the electrode gradually becomes narrower. But under the effect of fringe electric field, most of the charges are accumulated at the electrode edge between the driving electrode and the sensing electrode. It can be concluded that, when the number of electrode pairs is more, there are more areas where charges are concentrated, which makes the fringing electric field effect stronger. And when the number of electrode pairs is large, the charge distribution is more uniform macroscopically, so the electric field distribution is also more uniform, which means better linearity during measurement.

**Figure 6:**
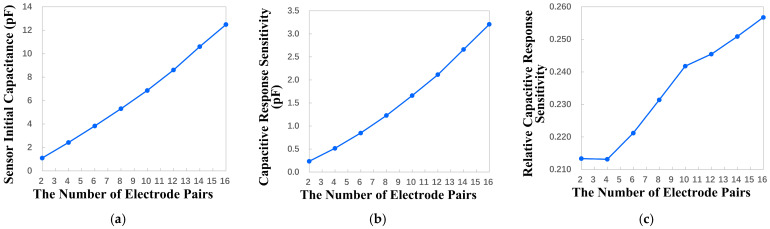
The influence of the number of electrode pairs variation on key performance indicators. (**a**) Sensor initial capacitance change. (**b**) Capacitive response sensitivity change. (**c**) Relative capacitive response sensitivity change.

Under the condition of fixed detection area and 50% metal coverage, the variation range of the number of electrode pairs is set to be 2 to 16. Through the parametric sweep, the relationship between the key performance indicators and the number of electrode pairs is obtained, as shown in Figure 6. The results show that the increase in the number of electrode pairs brings a great improvement to the overall performance of the sensor. The sensor’s initial capacitance increases from 1.1 pF to about 12.5 pF, which is a 12-fold increase. The capacitive response sensitivity of the sensor increases from 0.2 pF to 3.2 pF, which is a 16-fold increase. The relative capacitive response sensitivity increased with the increase in the number of electrode pairs, but the degree of change is small, from 0.21 to 0.25. The specific change in its value is shown in Table 1.

For the above reason, considering these three key performances comprehensively, the number of electrode pairs should be increased as much as possible under the fixed detection area and metal coverage.

#### 4.2.5. Influence of the Electrode Thickness on Key Performance

**Figure 7:**
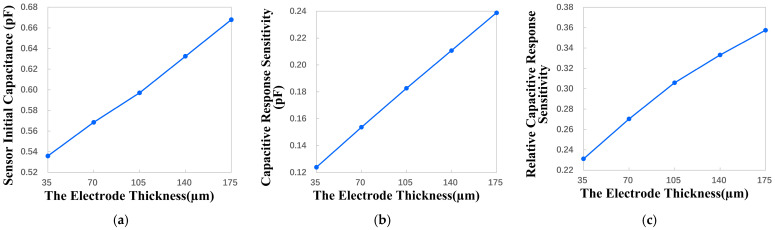
The influence of the electrode thickness variation on key performance indicators. (**a**) Sensor initial capacitance change. (**b**) Capacitive response sensitivity change. (**c**) Relative capacitive response sensitivity change.

According to the processing technology selected in this study, the minimum electrode thickness that can be machined is 35 μm, and the maximum electrode thickness that can be machined is 175 μm. Therefore, the variation range of the electrode thickness is set to be 35 μm to 175 μm with a step size of 35 μm. Through the parametric sweep, the relationship between the key performance indicators and the electrode thickness is obtained, as shown in Figure 7. The results show that the improvement in sensor performance brought by increasing electrode thickness is very small. When the electrode thickness is increased from 35 μm to 175 μm, the thickness becomes five times that of the original, but the increase in the initial capacitance, capacitance response sensitivity and relative capacitance response sensitivity is less than two times.

**Figure 8:**
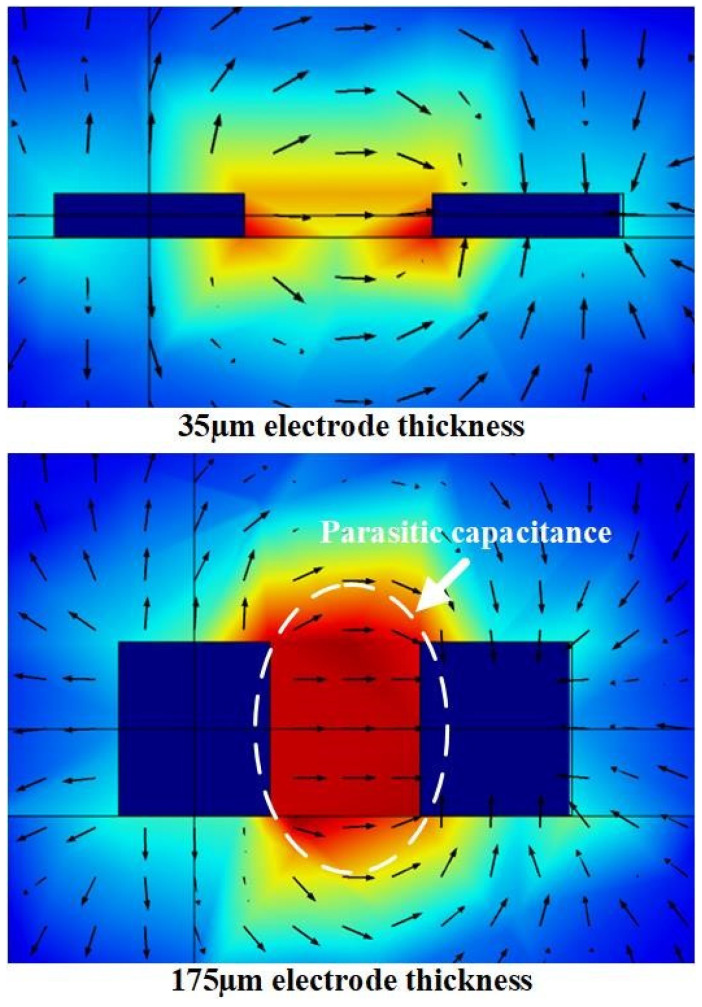
Distribution of electric field lines with different electrode thicknesses.

With the change in electrode thickness, the change in electric field line distribution is shown in Figure 8. According to the results, it can be concluded that, when the thickness of the electrodes is thicker, there are more electric field lines distributed in parallel between the electrodes, which means that more parasitic capacitances are introduced. The electric field line distribution when the electrode is thicker is also more scattered than when the electrode is thinner, which introduces nonlinear terms in the measurement. And when the thickness of the designed electrode is thicker, the consumption of consumables and the manufacturing cost of the sensor increase accordingly, and the economic benefit becomes worse.

For the above reason, the electrode thickness should be as thin as possible, such as 35 μm.

### 4.3. Sensor Prototype Design

#### 4.3.1. Sensor Prototype Design and Simulation

On the basis of PCB processing technology, according to the optimization direction of sensor structure parameters obtained from the research, this manuscript uses SOLIDWORKS 2023 software to design two sensor prototypes and the basic parameter information is shown in Table 2.

The two sensor prototypes are designed with different intentions. Prototype 1 is designed to verify the feasibility of the detection principle, so prototype 1 has a larger detection area and more electrode pairs. Prototype 2 increases the distance between the detection area and the conductive interface relative to prototype 1. Prototype 2 avoids the problem of short circuits between electrodes when measuring conductors, and it has better application capabilities.

The two sensor prototypes use the same design for some attributes. According to the investigation results in Section 4.2.1, the metal coverage is designed to be 50%. There are no special requirements for substrate material and solder mask material, so the manufacturer’s default substrate material (FR-4 glass fiber board) and default solder mask material (photosensitive ink) are selected, and their dielectric constants are 4.5 and 4.6, respectively. The thickness of the substrate should follow the principle of being as thin as possible, so the thickness of 1.5 mm is chosen in this manuscript. The electrode thickness is selected as 0.035 mm, which is the minimum thickness that can be processed by the manufacturer. This meets the design requirement for a minimal electrode thickness specified in Section 4.2.5. For the two-layer PCB, the processing limit of the minimum line width of the copper layer is 0.127 mm. In order to avoid the uncertainty of the processing quality of the machine under the limit processing capacity, the electrode width is selected to be 0.152 mm, which is close to the processing limit. The sensor has more electrode pairs in the detection area.

The SOLIDWORKS models of the two sensor prototypes are imported into COMSOL Multiphysics for overall finite element simulation analysis. The feasibility of the scheme is verified by calculating the three performance indicators of the initial capacitance, capacitance response sensitivity and relative capacitance response sensitivity of the two prototypes. The simulation results are shown in Table 3. The simulation results show that the initial capacitance and capacitance response sensitivity of the two prototypes are in the pF order. The two prototypes both achieve the expected design goals and are feasible.

#### 4.3.2. Physical Production of Sensor Prototype

The rationality of the sensor prototype design is verified by finite element analysis. Altium Designer 19 software is used to design the PCB layout of two sensor prototypes. The attribute parameters of the two prototypes are consistent with those in Table 2. Due to the simple structure and small area of the designed PCB prototypes, the cost of these micro-moisture sensor prototypes is low. The PCB layouts and physical prototypes of the two sensors are presented in the Supporting Information.

## 5. Design and Fabrication of Switched Capacitor Interface Circuit

In practical applications, there are many disturbances that affect the circuit, including channel charge injection effect, clock feedthrough effect and offset voltage of integrated operational amplifiers. They affect the accuracy, linearity, and sensitivity of the sensor. Therefore, this work chooses to design the sensor interface circuit based on the switched capacitor principle, in which a CMOS transmission gate switch and a correlated double sampling technique are used.

The overall structure of the switched capacitance sensor interface circuit includes the clock pulse generator, the correlated double sampling switched capacitor circuit, the signal conditioning circuit and the low-pass filter. The above modules are integrated, a standard capacitor is incorporated to represent the sensor’s equivalent sensitive capacitance, and the whole interface circuit is simulated and verified. The simulation results show that the input–output correlation coefficient of the interface circuit reaches 0.99999, and the scale factor is 10.1354 mV/pF. The simulation results show that the interface circuit is feasible in principle.

**Figure 9:**
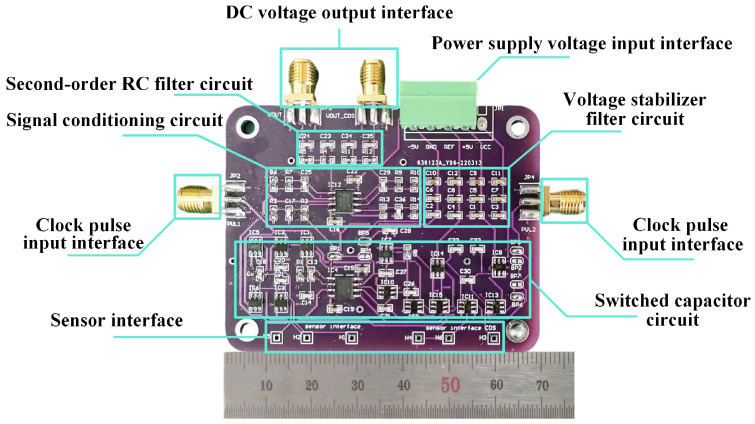
Prototype of the switched capacitor interface circuit for moisture detection.

According to the above design scheme, the PCB layout design of the interface circuit is completed. On the interface circuit prototype, the output interface of the switched capacitor circuit that does not use correlated double sampling is integrated, which can be compared with the output of the switched capacitor circuit that uses correlated double sampling. The effect of the correlated double sampling technique can be verified at the same time. Breakpoints between each module are added to the PCB layout, which can realize sub-module debugging and improve debugging efficiency. The SubMiniature version A Connector (SMA) is selected as the input and output interface of the circuit, which can effectively reduce the influence of environmental noise on the signal quality. The PCB after soldering is shown in Figure 9. This design can realize high-precision signal detection and low-cost interface circuit development.

## 6. Test Experiment and Result Analysis

The sensor prototype is tested first, and then the sensor prototype and the interface circuit are tested systematically. Through the above test, the correctness and rationality of the design scheme are verified and the performance index of the moisture detection system is evaluated. A dedicated test platform is constructed for the experimental tests, comprising an LCR digital bridge, a signal generator, a regulated power supply and a four-channel oscilloscope; the detailed setup of the test platform is provided in the Supporting Information.

### 6.1. Sensor Prototype Test

For sensor prototype 1 and prototype 2, five prototypes are fabricated for each design. In order to reduce the influence of processing error on the test, the initial capacitance of the ten prototypes is measured using an LCR digital bridge, and the average value and standard deviation are calculated. As shown in Table 4, the initial capacitance values of the five products of sensor prototype 1 fluctuate between 54.555 pF and 56.231 pF, the average value of the initial capacitance is 55.404 pF, and the standard deviation is 0.6822 pF. The initial capacitance of prototype 1 calculated by finite element analysis in Section 4.3 is 50.543 pF, which is close to the measured value. The initial capacitance values of the five products of the sensor prototype 2 fluctuate between 11.870 pF and 12.335 pF, with an average value of 12.0324 pF and a standard deviation of 0.1935 pF. And the simulated finite element simulation value is also close to the measured value. This shows that it is effective and reasonable to apply the finite element analysis method to the sensor design.

Because the essence of moisture detection is to detect the water mass in air, soil and other media, this paper uses the droplet device to detect the change in water mass in the detection area of the micro-moisture sensor to simulate moisture changes. According to the experiment, the mass m of each water droplet is 18 mg.

The principle of the capacitive moisture sensor is to convert the change in water mass in the detection area into the change in equivalent sensitive capacitance; it is necessary to measure the linearity and sensitivity of the input–output relationship of the micro-moisture sensor prototype. The water drop test experiment of the two sensor prototypes steps are as follows: For prototype 1, its detection area is larger and can carry more water mass, so five water droplets are added incrementally until the detection area is fully covered with water. After dripping five water droplets each time, the equivalent sensitive capacitance value is measured by the LCR digital bridge after the stabilization time of 1 min. Similarly, for prototype 2, the detection area is small, so only one drop of water is added each time until the water covers the detection area. Also, wait for 1 min stabilization time before measuring.

**Figure 10:**
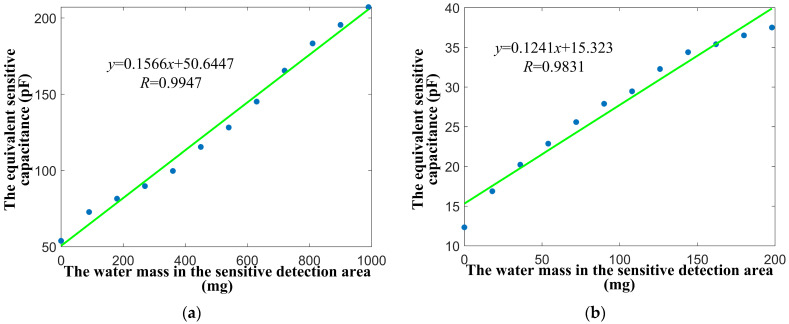
Experimental test results of sensor prototype water droplets. (**a**) Prototype 1. (**b**) Prototype 2.

The original data from the water drop tests of the two sensor prototypes are linearly fitted, with the results presented in Figure 10. For the sensor prototype 1, the correlation coefficient R between the equivalent sensitive capacitance and the water mass in the sensitive detection area is 0.9947, and the sensitivity is 0.1566 pF/mg. For sensor prototype 2, the correlation coefficient R is 0.9831, and the sensitivity is 0.1241 pF/mg. The test results show that the two sensor prototypes both have good linearity and sensitivity.

### 6.2. Overall Test and Performance Evaluation

Compared with sensor prototype 1 and sensor prototype 2, the difference in the linear correlation coefficient (ΔR) between the two prototypes is only 0.0116, with a slight difference in sensing sensitivity of 0.0325 pF/mg. These differences are small, but the error of prototype 2 produced in the same batch is smaller. In order to ensure the consistency of the prototype in mass production, and because of the large distance between the detection area and the conductive interface, prototype 2 has a greater application prospect. Therefore, the detection system composed of the sensor prototype 2 and the switched capacitor interface circuit using correlated double sampling is experimentally tested and the performance indicators are evaluated.

Nonlinear problems generally exist in various sensors, and a practical solution is to select a measurement range with superior linearity. According to the test results of the sensor prototype 2 in Section 6.1, it can be seen that, when there is water in the detection area at the beginning and when the water almost covers the entire detection area at the end, the sensor output shows obvious nonlinearity. When the water mass in the detection area is in the range of 18 mg to 162 mg, the output response linearity of the sensor is better. Therefore, it is determined to conduct experiments within this range. By using the droplet device, the water mass in the detection area is increased drop by drop, and the stabilization time of 1 min is waited for. Five measurements are performed using an oscilloscope, and the average value of these five measurements is obtained. These measurement data and calculation results form a data set. The data set is used as the calibration set to fit the mathematical relationship between the water mass in the detection area and the output DC voltage of the interface circuit, and to calibrate the sensitivity and linearity of the system.

**Figure 11:**
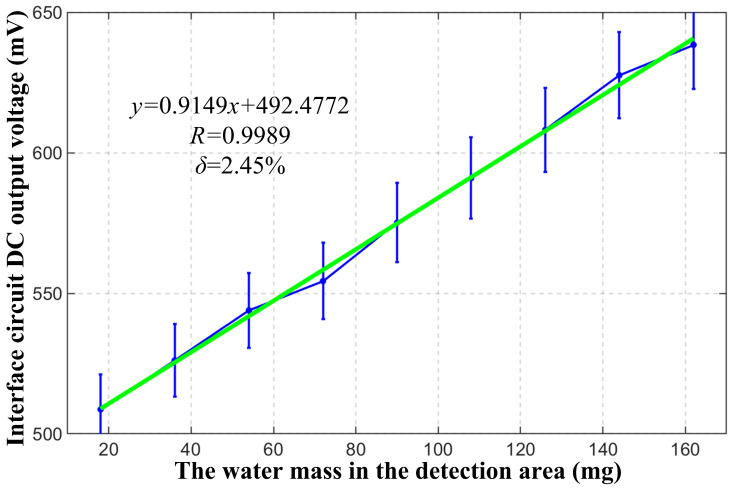
Analysis of linearity and sensitivity results of moisture detection system.

The original data for this calibration set are recorded, and the data are analyzed by linear fitting using MATLAB R2023b. The derived results are illustrated in Figure 11. The fitting result of the linear relationship between the water mass in the detection area and the amplitude of the DC output voltage of the system is as follows:

(14) y=0.9149x+492.4772.

The correlation coefficient R between the water mass in the detection area and the output amplitude of the DC voltage of the system is 0.9989, and the sensitivity is 0.9149 mV/mg. A nonlinear error of 2.45% is calculated as the ratio of the maximum deviation between the fitted straight line and the actual response curve of the moisture detection system to its full-scale output.

**Figure 12:**
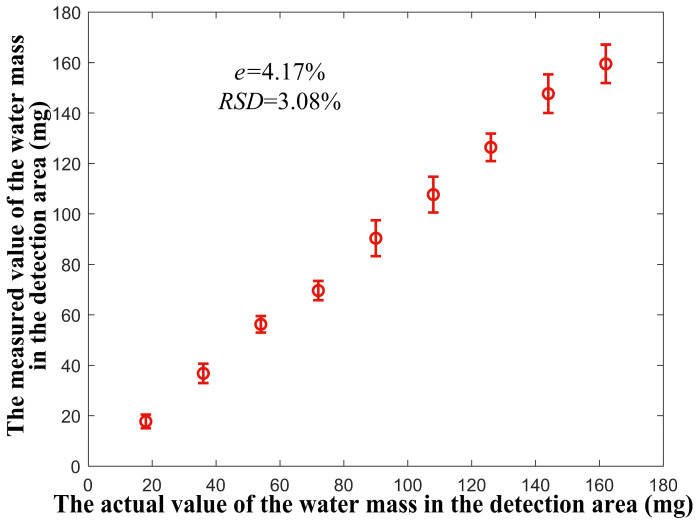
Analysis of accuracy and precision performance of moisture detection system.

In order to evaluate the accuracy and precision of the moisture detection system, the experiment is again carried out in the range of water mass in the detection area from 18 to 162 mg. By repeating the above experimental steps, a new data set is obtained. The data set is used as the test set to evaluate the accuracy and precision of the moisture detection system. By substituting the data of the test set into Equation (14), the measured value of water mass corresponding to the actual value of water mass in each detection area can be obtained. The relationship between the actual value and the measured value of the water mass in the detection area is shown in Figure 12. Comparing the actual value of water mass with the measured value, it can be calculated that, within this range, the maximum relative error e=4.17%, and the maximum relative standard deviation RSD=3.08%.

In order to make the experimental results conform to the standard of moisture measurement, the experiment on the change in water droplet and moisture is carried out. According to the experimental results, the change in air relative moisture caused by each drop is 0.1263%RH/mg. Therefore, the sensitivity of the sensor is 1.2403 pF/%RH, and the sensitivity of the moisture detection system is 7.2439 mV/%RH. The comparison with the research work of the fringe electric field sensor in recent years is shown in Table 5.

## 7. Conclusions

Based on theoretical analysis and experimental verification, the influence of structural parameters on the performance of the fringe electric field-based micro-moisture sensor is discussed in this paper. It can be seen from the discussion that the metal coverage rate of 50% is the most appropriate, the substrate thickness should be reduced as far as possible, and the electrode thickness should be as thin as possible. The number of electrode pairs should be increased as much as possible under the fixed detection area and metal coverage. Proposed prototypes achieved sensitives of 0.1566 pF/mg and 0.1241 pF/mg. However, the results for prototype 1 show that increasing the number of electrode pairs increases the initial capacitance, but yields only limited improvements in the sensor’s linearity and sensitivity, and it is difficult to manufacture the consistency of the same batch of sensors. The sensor prototype 2 with fewer electrode pairs not only ensures linearity and sensitivity, but also ensures the consistency of manufacturing the same batch of sensors, so the sensor prototype 2 is more suitable for mass manufacturing moisture detection systems. After calculation, the linearity of the sensor is 0.9947, the sensitivity is 1.2403 pF/%RH, the linearity of the moisture detection system is 0.9989, and the sensitivity is 7.2439 mV/%RH. These experimental results prove the effectiveness and practical value of the structural optimization, and further lay a solid foundation for the system-level application and industrialization of the fringing field micro-moisture sensor.

The structural optimization approach proposed in this study is also applicable to other types of fringing electric field sensors for detection applications fabricated via PCB processing, which can be extended, such as water droplets sensors. In addition, hygroscopic sensitive materials can be coated in the sensor detection area for the optimized moisture sensor. This can be achieved to further improve the response sensitivity of the moisture sensor. This optimized sensor also holds promising system-level application prospects for practical deployment [34,35]. Benefiting from its PCB process compatibility, low cost and high batch consistency, it can be easily integrated into intelligent moisture detection systems for agriculture, industry, aerospace and other key fields, and supports large-scale industrial deployment and real-world application.

## Figures and Tables

**Table 1:** The relationship between the relative capacitive response sensitivity and the number of electrode pairs.

Electrode Pairs	2	4	6	8	10	12	14	16
Relative capacitive response sensitivity	0.2133	0.2131	0.2212	0.2314	0.2417	0.2454	0.2509	0.2567

**Table 2:** Sensor prototype basic attribute table.

Basic Properties	Prototype 1	Prototype 2
size	25 mm × 25 mm	83 mm × 27 mm
number of electrode pairs	35	15
metal coverage	50%	50%
substrate material/dielectric constant	FR-4 glass fiber board/4.5	FR-4 glass fiber board/4.5
substrate thickness	1.5 mm	1.5 mm
electrode thickness	35 μm	35 μm
electrode width	0.152 mm	0.152 mm
solder mask material/dielectric constant	photosensitive ink/4.6	photosensitive ink/4.6

**Table 3:** Sensor prototype performance simulation results.

Key Performance Indicators	Prototype 1	Prototype 2
Initial Capacitance/pF	50.543	10.728
Capacitive Response Sensitivity/pF	8.587	1.948
Relative Capacitive Response Sensitivity	16.99%	18.16%

**Table 4:** The initial capacitance test results of the two sensor prototypes.

**Parameter**	**Prototype 1**	**Prototype 2**
Initial capacitance of prototype No. 1/pF	55.128	12.335
Initial capacitance of prototype No. 2/pF	55.963	11.883
Initial capacitance of prototype No. 3/pF	56.231	11.968
Initial capacitance of prototype No. 4/pF	54.555	11.870
Initial capacitance of prototype No. 5/pF	55.143	12.106
Average value of initial capacitance/pF	55.404	12.032
Standard deviation of initial capacitance/pF	0.6822	0.1935
Initial capacitance finite element simulation results/pF	50.543	10.728

**Table 5:** Comparative results of research work on fringe electric field sensor.

Key Performance Indicators	This Paper	Reference [17]	Reference [20]	Reference [28]	Reference [29]	Reference [31]
Sensor linearity	R = 0.9947	0.9763 < R < 0.9774	R ≈ 0.99	R = 0.872	R = 0.985	R < 0.98
Sensor sensitivity	1.2403 pF/%RH	265 kHz/%RH	≈1.5 pF/%RH	0.1156 pF/%RH	≈0.35 pF/%RH	40 fF/mm
Consistency	High	Not available	High	Not available	Medium	Medium
Sensing layer	Copper layer	Graphene oxide	Copper layer	Copper layer	Copper layer + polymer dielectric	Copper layer
Size	25 mm × 25 mm/ 83 mm × 27 mm	Not available	Custom multi-frame	96.5 mm × 43.2 mm	20 mm × 20 mm	35 mm × 35 mm
Cost	Low	High	Medium	Medium	Low	Low

## Data Availability

Dataset available on request from the authors.

## Supplementary Materials

The following Supporting Information can be downloaded at: https://www.mdpi.com/article/10.3390/mi17030388/s1, Figure S1. PCB layout and physical prototype of Sensor Prototype 1; Figure S2. PCB layout and physical prototype of Sensor Prototype 2; Figure S3. Experimental test platform for the moisture detection system.
