# Supplementary material for: Enhanced Fringing Field Micro-Moisture Sensor with Elements Optimization
Source: Micromachines (Basel). 2026 Mar 23;17(3):388. doi: 10.3390/mi17030388 (PMC13028875; doi:10.3390/mi17030388)

Supplementary Materials

Figure S1

Title: PCB layout and physical prototype of Sensor Prototype 1

Caption: The PCB layout (left) and actual physical prototype (right) of the interdigitated fringing field moisture sensor Prototype 1, with the scale bar marked as 10 mm and 20 mm for reference.

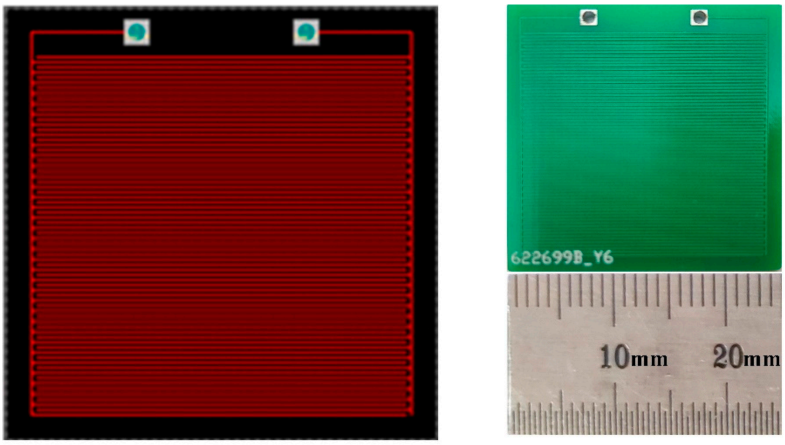

PCB layout of prototype 1 **Physical map of prototype 1**

Figure S2

Title: PCB layout and physical prototype of Sensor Prototype 2

Caption: The PCB layout (left) and actual physical prototype (right) of the interdigitated fringing field moisture sensor Prototype 2, with the scale bar marked from 10 mm to 80 mm for reference.

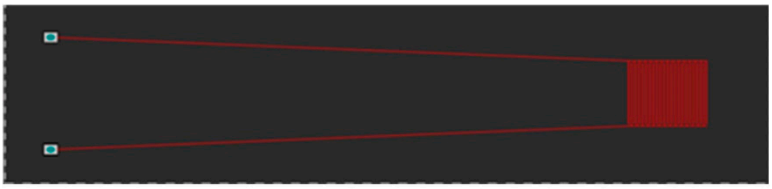

PCB layout of prototype 2

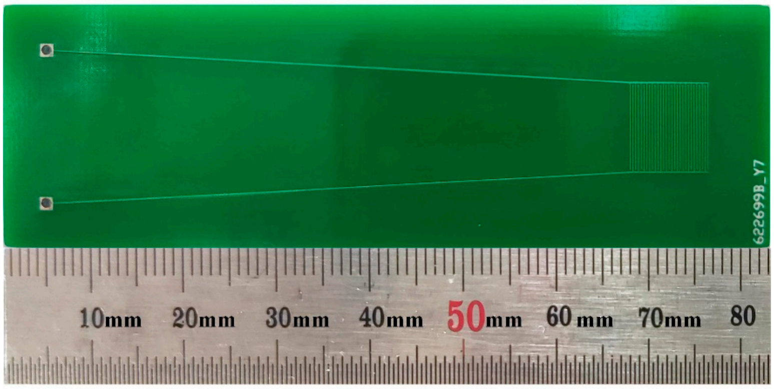

Physical map of prototype 2

Figure S3

Title: Experimental test platform for the moisture detection system

Caption: The complete test platform consists of an LCR digital bridge, a signal generator, a regulated power supply, a four-channel oscilloscope, the sensor prototype and the interface circuit prototype, which is used for the performance test of the sensor and the entire detection system.

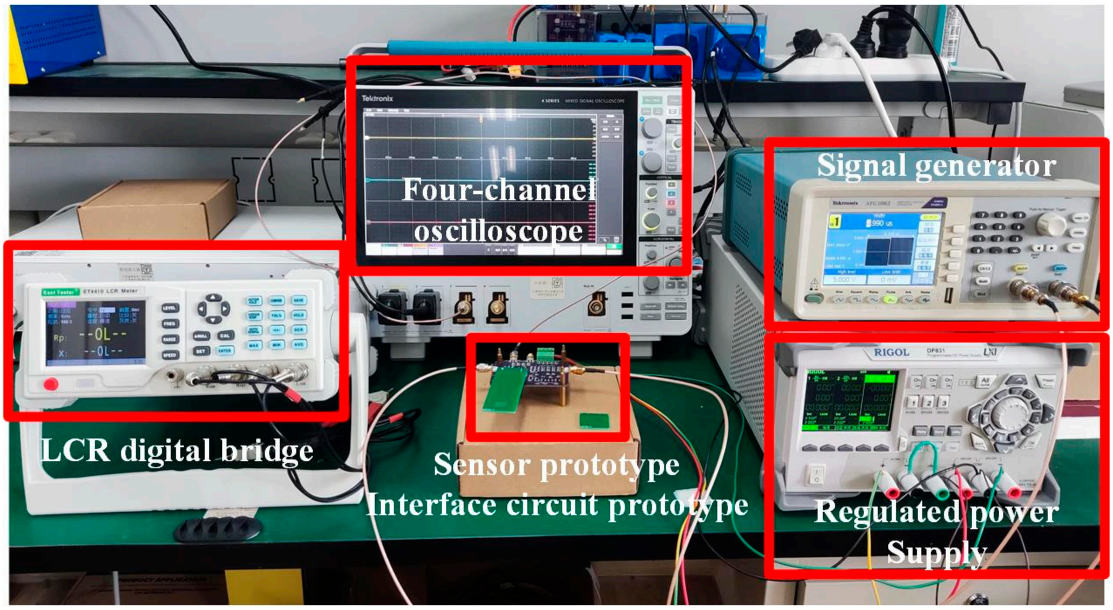

Supplement: Supplementary file 1 [file micromachines-17-00388-s001.zip › micromachines-4167669-supplementary.pdf]
